# Analysing the blood-stemming effect of Ankaferd Blood Stopper in medulla spinalis surgery

**DOI:** 10.3906/sag-2001-249

**Published:** 2020-06-23

**Authors:** Dursun TÜRKÖZ, Cem DEMİREL, Hüseyin SATALOĞLU, Cengiz ÇOKLUK

**Affiliations:** 1 Department of Neurosurgery, Samsun Training and Research Hospital, Samsun Turkey; 2 Department of Neurosurgery, Faculty of Medicine, Ondokuz Mayıs University, Samsun Turkey

**Keywords:** Ankaferd Blood Stopper, haemostasis, spinal cord

## Abstract

**Background/aim:**

The aim of this study was to investigate the possible toxicity of the Ankaferd Blood Stopper (ABS) on the neural system.

**Materials and methods:**

Thirty Sprague Dawley rats were randomized into ABS (n: 15) and control (n: 15) groups. Following the anaesthetic induction, total laminectomy was performed to the lower thoracic, and upper lumbar areas in both groups and medulla spinalis was exposed. Two myelotomies were performed on the medulla spinalis. One millilitre ABS was applied to the incision site in the ABS group, and one millilitre 0.9% saline solution was applied in the control group. Rats were observed for 15 days regarding general behaviour, neurological signs, mobility, and signs of infection. Sixteen days later, all rats were decapitated under anaesthesia. Medulla spinalis was removed en bloc from all rats and was stained with Heamatoxylin & Eosin and luxol fast blue.

**Results:**

There was no significant difference between the ABS group and the control group regarding oedema, gliosis, the intensity of inflammatory cells, the presence of neuronal degeneration, neuron counts, and myelin degeneration.

**Conclusion:**

No clinical or histopathological evidence for the neurotoxic effect of the ABS was observed in the present study. Our findings might precipitate the use of ABS on human subjects regarding medulla spinalis surgery.

## 1. Introduction

Haemostasis is described as the physiological systems which are necessary for stemming the bleeding and ensuring vascular integrity without blocking the blood flow. In terms of surgical procedures, adequate haemostasis provides many advantages in the postoperative period. Mechanical methods, bipolar cautery, oxidized cellulose, collagen, and gelatine are commonly employed for bleeding control. However, each of these methods has its risks and advantages. Ankaferd Blood Stopper (ABS) is a relatively new and effective method for bleeding control. ABS is used in traditional Turkish medicine and is prepared by mixing 5 herbal ingredients (*Glycyrrhiza glabra, Vitis vinifera, Alpina officinarum, Urtica dioica, Thymus vulgaris*) in different proportions. ABS affects blood proteins (mainly fibrinogen) and erythrocytes, and acts rapidly by forming a protein network. ABS is a preferably haemostatic agent which is easy to apply and does not damage healthy tissue. 

The topical haemostatic efficacy of ABS has been previously tested in animals with normal and defective haemostasis [1,2]. Physiological cell-based coagulation could be clinically managed via topical ABS applications to prevent and treat bleeding in many distinct clinicopathological states [3,4]. Neither local nor systemic adverse effects and/or toxicity have been observed in association with experimental and anecdotal topical application of ABS.

The neurotoxic effects of ABS on neural tissue are yet to be investigated. Therefore, the neurotoxic effect of ABS was studied histopathologically in the present animal study.

## 2. Materials and methods

### 2.1. Experimental animals

Prior to the study, ethical approval was obtained from Animal Studies Local Ethics Committee of Ondokuz Mayıs University with number 2008-37. Sprague Dawley male rats (16–20 weeks and 300–400 g) were used. A total of 30 rats were randomized into 2 groups as the ABS group (n: 15) and the control group (n: 15). Each group was kept in separate cages, in their natural environments, without food and water restrictions until 12 h prior to the experiment. 

### 2.2. Procedure

Each rat was sedated by using 10 mg/kg xylazine (Rompun, Bayer) peritoneally. For anaesthesia, 80 mg/kg ketamine hydrochloride (Ketalar, Parke Davis) was applied intraperitoneally. Rats were placed on the surgery table following the anaesthesia. Then the surgical site was shaved and was cleaned with a 10% povidone-iodine solution. A 3-cm incision was applied to the lower thoracic and upper lumbar area. Then paravertebral muscle fascia was cleared. Paravertebral muscles were removed accordingly, and a 2-level total laminectomy was performed. Dura and arachnoid were cleared, and a 2-cm medulla spinalis region was revealed. Two incisions (3-mm length and 2-mm deep) were applied on medulla spinalis. One of the incisions was on the midline (fascicules and surroundings), and the other one was on lateral (posterior horn and surroundings). In the experimental group, 1 mL ABS was applied to the incision area, while 0.9% saline solution was used for the control group. Incisions were closed in accordance with the anatomical layers. The rats were placed in their preheated cages. The rats awakened in those cages and were received 2 mg/kg paracetamol for 3 days. Rats were followed for 15 days regarding to general behaviour, neurological findings, mobility, and infection. The rats were excluded from the study if the following signs were observed: abnormal posture and motor deficits, redness in the incision area, infection symptoms such as dripping, or decrease in the food/liquid intake. Sixteen days later, rats were decapitated under anaesthesia; medulla spinalis were removed as en bloc from the upper and lower borders of the incision sites. 

### 2.3. Histopathologic analysis

Medulla spinalis tissue was fixed in the formalin solution. Transverse sections were extracted from medulla spinalis tissues and were embedded in paraffin blocks. Five-micron sections were obtained from paraffin blocks and were stained with haematoxylin and eosin stain (H-E) and luxol fast blue stain. A pathologist who was blind to the groups performed the histopathological analysis by using a light microscope. Oedema, gliosis, neuronal degeneration, and inflammatory cell concentration were evaluated in H&E stained sections, and myelin degeneration was evaluated in luxol fast blue-stained sections. Findings were scored as none (0), mild (1), and significant (2). In addition, neurons with a nucleus that are investigated by using a 40 × 40 magnifier were counted [5,6,7].

No hazardous materials to human or environmental health were used in the present study. Dead animals and other materials were stored in special bags and disposed in special waste containers. 

### 2.4. Statistical analysis

Statistical analysis was performed by using SPSS for Windows version 15.0 (SPSS Inc., Chicago, IL, USA). Mann–Whitney U test was employed to perform between group comparisons. P-value of <0.05 was accepted as statistically significant. 

## 3. Results

None of the rats were excluded from the study due to exclusion. The distribution of the findings from the histopathological evaluation is shown in Table 1 and Table 2. 

**Table 1:** Histopathological findings in ABS group.

Rats	Neuron count	Oedema	Gliosis, reactive astrocytoma	Neuronal degeneration	Inflammatorycell	Myelin degeneration
ABS1	11	0	0	1	0	0
ABS2	10	0	1	0	1	0
ABS3	5	0	0	1	1	0
ABS4	12	0	0	0	0	0
ABS5	11	0	0	0	0	0
ABS6	9	0	0	0	0	0
ABS7	14	0	0	0	0	0
ABS8	11	1	0	0	0	0
ABS9	9	1	0	1	2	0
ABS10	3	1	0	0	1	1
ABS11	6	1	1	0	0	0
ABS12	7	1	0	1	2	0
ABS13	13	0	0	0	0	0
ABS14	11	1	1	0	0	0
ABS15	13	0	0	0	0	0

**Table 2:** Histopathological findings in control group.

Rats	Neuron count	Oedema	Gliosis, reactive astrocytoma	Neuronal degeneration	Inflammatory cell	Myelin degeneration
Control1	13	0	0	0	0	0
Control2	9	1	1	0	0	1
Control3	14	1	0	0	0	0
Control4	9	0	0	0	0	0
Control5	7	1	0	1	2	0
Control6	11	1	0	0	1	0
Control7	6	1	0	1	1	0
Control8	4	1	0	0	1	0
Control9	22	1	0	0	1	0
Control10	19	0	0	0	1	0
Control11	16	0	0	0	0	0
Control12	14	0	0	0	1	0
Control13	14	0	0	0	1	0
Control14	4	1	0	1	1	0
Control15	6	0	0	0	0	0

There was no statistical difference between groups related to neuron count (P > 0.05). A mild myelin degeneration was observed in 1 rat in each group (P > 0.05). 

Mild oedema was detected in 6 rats (40%) in the ABS group, and in 8 rats (53%) in the control group (P > 0.05), no severe oedema was found in none of the groups. 

Mild neuronal degeneration was detected in 4 rats (26%) in the ABS group, and in 3 rats (20%) in the control group (P > 0.05), no severe neuronal degeneration was detected in none of the groups. 

Mild inflammatory cell density was found in 3 rats (20%) in the ABS group, and in 8 rats (52%) in the control group (P > 0.05) Severe inflammatory cell density was detected in 2 rats (13%) in the ABS group, and 1 rat (6.5%) in the control group (P > 0.05).

Mild gliosis was developed in 3 rats (20%) in the ABS group, and 1 rat (6.5%) in the control group (P > 0.05). The comparison of 2 groups is given in Table 3. 

**Table 3:** Comparison of 2 groups.

	Control			ABS			P
	None	Mild	Severe	None	Mild	Severe	
Oedema	7	8	-	7	8	-	P > 0.05
Gliosis, reactive astrocytoma	12	3	-	14	1	-	P > 0.05
Neuronal degeneration	11	4	-	12	3	-	P > 0.05
Inflammatory cell	10	3	2	6	8	1	P > 0.05
Myelin degeneration	14	1	-	14	1	-	P > 0.05

## 4. Discussion

Excessive blood loss is one of the most critical problems in spinal surgery. Inadequate haemostasis and uncontrolled bleeding might lead to many complications from neurologic function loss to death. Moreover, frequent use of anticoagulant drugs such as aspirin in the general population worsens the problem. Many different approaches, including compression, topical haemostatic drugs, sutures, and ligation clips are used for blood stemming [8,9]. However, compression or ligation approaches are not suitable methods for spinal surgeries as the target tissues are deep-placed and fragile [10,11]. Thus, topical haemostatic agents are preferred for blood stemming. ABS is a relatively new topical haemostatic agent for blood stemming. The aim of the present study was the investigate the neurotoxic effect of ABS on neural tissue. Our results showed that ABS has very few neurologic toxicities for spinal surgeries. 

Gelatine (Gelfoam) and oxidized cellulose are the most commonly used topical haemostatic agents, and they have been used for nearly 40 years in neurosurgery [11,12]. However, studies reported that oxidized cellulose or gelatine expands into the intervertebral foramen and compresses the spinal cord, leading paraplegia or imitating tumour recurrence or abscess in computerized tomography or magnetic resonance imaging in the postoperative period [12–16]. No deficits were observed in motion or motor abilities following ABS application in the present study. According to our results, due to the liquid form of ABS, no compression effect occurred on the spinal cord. 

A comparison of the topical haemostatic agents was reported in the literature. Karakaya et al. compared regenerated oxidized cellulose (Surgicel) and ABS in the experimental liver laceration model and reported that both agents have similar effects on blood stemming [17]. These authors also observed no difference in inflammatory cell intensity as in the present study. Huri et al. reported similar results following ABS application on renal trauma [18]. Moreover, many other beneficial aspects of ABS such as having inhibitory activity on both gram-positive and gram-negative bacteria [19], showing a rapid haemostatic response, and inducting a protein web formation which is stimulating erythrocyte aggregation in seconds [20] makes ABS a preferable topical haemostatic agent. 

Bone wax is often preferred in lumbar spinal surgeries due to its tamponade characteristics on bone tissue. However, it does not present haemostatic characteristics. Moreover, side effects such as granuloma, allergic reaction, infection, epistaxis, and compression were reported due to bone wax application [21]. Other approaches, such as bipolar cautery for blood stemming, have other disadvantages, such as thermal injury risk [10,22]. Diamantis et al. reported severe clinical and histologic complications such as coagulation necrosis due to bipolar cautery applications in rabbits [23]. It seems that the ABS approach has advantages over other methods in spinal surgery. Moreover, no serious toxicity was observed in neural structures in the present study. However, future comparison studies are needed to reach a consensus. 

Rats were sacrificed at the day 16 in the present study. However, there is no consensus about how many days are needed following the ABS application. The follow-up days are ranged from 7 to 42 days in the reported literature [1,24]. Orhan et al. used a 2-week follow-up similar to the present study [25].

According to our knowledge, the present study is the first study investigating the utility of ABS for medulla spinalis surgery. The mild side effects, such as neuronal degeneration, gliosis, oedema, and inflammatory cell intensity might be resulted from the surgery itself in the present study. However, lack of a placebo group is the limitation of the present study. 

In conclusion, ABS is an herbal mixture that showed good haemostatic properties and no severe side effects for spinal surgeries in animals. Our findings might precipitate the use of ABS on human subjects, but more research using more sensitive assessments such as electron microscopy and placebo groups are still needed.

## Conflict of Interest

Authors declare no conflict of interest.
